# Evaluation of clinical examination, ultrasonography, mammography, and magnetic resonance imaging for detection of axillary metastases in overweight and obese women with early-stage breast cancer: a systematic review and meta-analysis

**DOI:** 10.1590/0100-3984.2025.0057

**Published:** 2025-12-08

**Authors:** Carla Andries Cres Lyrio, Luis Otávio Zanatta Sarian, Rodrigo Menezes Jales

**Affiliations:** 1 Faculdade de Ciências Médicas da Universidade Estadual de Campinas (FCM-Unicamp), Campinas, SP, Brazil; 2 Hospital da Mulher Prof. Dr. José Aristodemo Pinotti - Caism/Unicamp, Campinas, SP, Brazil

**Keywords:** Ultrasonography, Mammography, Magnetic resonance imaging, Breast cancer, Obesity, Ultrasonografia, Mamografia, Ressonância magnética, Câncer de mama, Obesidade.

## Abstract

Accurate preoperative assessment of axillary lymph node status is essential for guiding treatment in early-stage breast cancer. Because clinical examination alone is often inadequate, imaging modalities such as axillary ultrasonography (AUS), mammography, and magnetic resonance imaging (MRI) are integral to axillary staging. Obese women with breast cancer have poorer oncologic outcomes than do their non-obese counterparts, which raises concerns about potential limitations in diagnostic performance due to a high body mass index (BMI). The objective of this study was to evaluate the diagnostic performance of clinical examination, AUS, mammography, and MRI in detecting axillary metastases in overweight and obese women with early-stage breast cancer. A systematic review and meta-analysis were conducted following the Preferred Reporting Items for a Systematic Review and Meta-Analysis of Diagnostic Test Accuracy guidelines. We included studies assessing the diagnostic accuracy of clinical and imaging modalities for detecting axillary metastasis in overweight and obese women. Methodological quality was assessed by using the Quality Assessment of Diagnostic Accuracy Studies 2 tool. Sensitivity and specificity data were extracted when available, and summary receiver operating characteristic curves were constructed. Nine studies met the inclusion criteria. The most frequently evaluated modality was AUS, which consistently demonstrated preserved diagnostic performance across weight groups; however, one retrospective cohort study reported that its negative predictive value decreases in parallel with increases in BMI. One study involving over 5,000 patients showed that the clinical examination is not significantly affected by the patient BMI. Mammography and MRI showed more variable results, with one study showing MRI performance potentially being impaired in overweight patients, although that study was rated as having a high risk of bias. Across studies, no substantial evidence supported the need for modifying diagnostic protocols based on BMI. Clinical examination and AUS continue to be reliable methods for axillary staging in overweight and obese women with early-stage breast cancer. Given one contradictory cohort study, negative AUS findings in obese patients should be interpreted with caution until standardized AUS criteria and prospective BMI-stratified studies are available. Further high-quality, prospective studies are needed in order to confirm these findings and to inform evidence-based refinements in staging protocols.

## Abstract (pt)

A avaliação pré-operatória precisa do *status* dos linfonodos axilares é fundamental para o planejamento terapêutico no câncer de mama em estágio inicial. Embora o exame clínico isolado seja frequentemente insuficiente, métodos de imagem como a ultrassonografia (US) axilar, a mamografia e a ressonância magnética (RM) desempenham um papel essencial no estadiamento axilar. Mulheres obesas com câncer de mama apresentam desfechos oncológicos piores em comparação às não obesas, o que levanta preocupações quanto a possíveis limitações no desempenho diagnóstico relacionadas ao aumento do índice de massa corporal (IMC). O objetivo deste estudo foi avaliar o desempenho diagnóstico do exame clínico, da USG, da mamografia e da RM na detecção de metástases axilares em mulheres com sobrepeso ou obesidade e câncer de mama em estágio inicial. Foi realizada uma revisão sistemática com metanálise conforme as diretrizes do *Preferred Reporting Items for a Systematic Review and Meta-Analysis of Diagnostic Test Accuracy*. Foram incluídos estudos que avaliaram a acurácia diagnóstica de métodos clínicos e de imagem para metástase axilar em mulheres com sobrepeso ou obesidade. A qualidade metodológica foi avaliada com a ferramenta *Quality Assessment of Diagnostic Accuracy Studies 2*. Quando disponíveis, foram extraídos dados de sensibilidade e especificidade, e construídas curvas *receiver operating characteristic* resumidas. Nove estudos preencheram os critérios de inclusão. A US axilar foi a modalidade mais frequentemente avaliada, demonstrando desempenho diagnóstico preservado nos diferentes grupos de peso; entretanto, uma coorte retrospectiva descreveu redução do valor preditivo negativo da US axilar com o aumento do IMC. Um estudo com mais de 5.000 pacientes mostrou que o exame clínico não foi significativamente influenciado pelo IMC. Mamografia e RM apresentaram resultados mais variáveis, com possível redução do desempenho da RM em pacientes com sobrepeso em um estudo classificado com alto risco de viés. De forma global, não houve evidência consistente para a necessidade de modificar protocolos diagnósticos exclusivamente com base no IMC. O exame clínico e a US axilar permanecem métodos confiáveis para o estadiamento axilar em mulheres com sobrepeso ou obesidade e câncer de mama em estágio inicial. À luz de um achado contraditório, resultados negativos na US axilar em pacientes obesas devem ser interpretados com cautela até que critérios padronizados deste exame e estudos prospectivos estratificados por IMC estejam disponíveis. Novos estudos prospectivos e de alta qualidade são necessários para confirmar esses achados e embasar possíveis refinamentos nos protocolos de estadiamento.

## INTRODUCTION

In the assessment of early-stage breast cancer with clinically negative axillary lymph nodes, sentinel lymph node dissection (SLND) is part of the conventional approach for detecting axillary metastasis^**(^1^)**^. For patients with three or more metastatic lymph nodes identified through SLND, the standard procedure includes axillary lymph node dissection (ALND) as a complementary procedure^**(^1^)**^. Notably, patients with only one or two metastatic lymph nodes may safely forgo ALND without compromising oncologic outcomes^**(^2^)**^.

However, clinical examination alone is inadequate for detecting subcentimeter lymph node metastases and has low specificity in differentiating between reactive and metastatic lymph nodes^**(^3^-^5^)**^. Therefore, the 9th edition of the American Joint Committee on Cancer staging manual recommends the use of imaging findings, in addition to lymphoscintigraphy, to increase the accuracy of axillary clinical staging^**(^2^)**^.

Although mammography is the standard method for breast cancer screening, its capacity to evaluate the axillary region is limited by its narrow field of view^**(^6^)**^. In addition, no standardized mammographic criteria exist to differentiate metastatic from non-metastatic lymph nodes^**(^6^)**^. Among noninvasive imaging modalities, axillary ultrasonography (AUS) stands out as the primary method for detecting axillary lymph node metastasis in newly diagnosed breast cancer patients^**(^7^,^8^)**^. The lower sensitivity of positron-emission tomography/computer tomography (PET/CT) and PET alone may be attributable to the lower spatial resolution of PET and the presence of artifacts on PET/CT fusion images^**(^9^)**^. In contrast, magnetic resonance imaging (MRI) shows superior performance for nodal evaluation^**(^8^)**^. Nonetheless, whether AUS should be replaced by these alternative methods remains a matter of debate^**(^8^)**^.

Over the past decade, AUS has gained attention as a noninvasive alternative to SLND in early-stage breast cancer because of its superior ability to detect extensive axillary disease^**(^10^-^14^)**^. Avoiding SLND could reduce the physical and psychological burden on patients^**(^11^)**^.

Obese women with breast cancer tend to have worse disease-free and overall survival than do their non-obese counterparts, even if receiving appropriate local and systemic treatment^**(^15^)**^. These outcomes are attributed to a combination of diagnostic, physical, psychosocial, biological, and therapeutic challenges^**(^15^)**^. Optimizing diagnostic and therapeutic strategies for obese women with breast cancer is therefore critical^**(^15^)**^.

**Figure 1:**
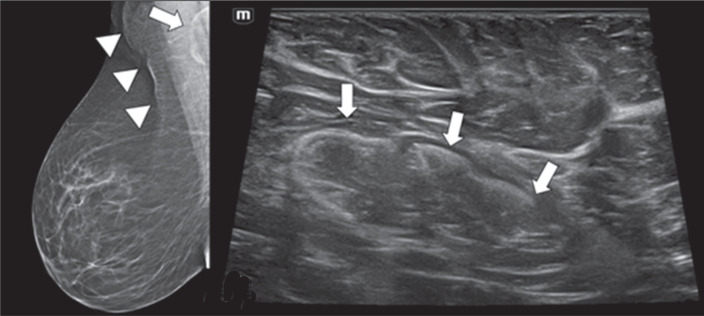
Mammogram and AUS of an obese patient diagnosed with contralateral breast cancer. A: Mammogram in right mediolateral oblique view showing impaired image quality due to redundant skin folds (arrowheads). Note the enlarged axillary lymph node with expansion of the hilum caused by hypodense fatty infiltration (arrow). B: Corresponding AUS image showing the same lymph node, with increased dimensions (3.3 cm × 1.2 cm) and a hilum with heterogeneous echotexture, potentially obscuring cortical boundaries. These features may limit the diagnostic performance of both modalities in the detection of axillary lymph node metastasis.

A high body mass index (BMI) may complicate axillary assessment by increasing lymph node dimensions due to fatty infiltration of the hilum (Figure 1), which may obscure subtle abnormalities and alter established morphological criteria for malignancy^**(^16^,^17^)**^. Obesity may also hinder proper patient positioning during mammography (Figure 1A) and MRI^**(^15^-^18^)**^, as well as compromising the clinical examination and ultrasound transmission due to thick subcutaneous fat in the axilla^**(^16^,^19^)**^, as illustrated in Figure 1B. It is noteworthy that fat infiltration in contralateral axillary lymph nodes on MRI has been associated with a higher likelihood of metastases in obese women, independent of tumor characteristics, whereas BMI itself has not been shown to be a determining factor^**(^20^)**^. Among obese patients, sentinel node detection rates tend to be lower and mapping failure rates tend to be higher^**(^21^-^23^)**^.

Given the importance of understanding how obesity affects preoperative axillary lymph node assessment, this systematic review and meta-analysis aimed to evaluate the diagnostic performance of clinical examination and of the main imaging modalities-mammography, ultrasonography, and MRI-in overweight and obese women with early-stage breast cancer.

## METHODS

This study adhered to the principles outlined in the Preferred Reporting Items for Systematic reviews and Meta- Analyses of Diagnostic Test Accuracy Studies (PRISMA-DTA) statement^**(^24^)**^. The protocol was registered on the International Prospective Register of Systematic Reviews platform (Protocol no. CRD42022315920; last updated May 2025). We searched the Cochrane, Embase, PubMed/Medline, Scopus, and Web of Science databases, from their inception to June 2022, limiting results to human studies and publications in English only. The search was updated in May 2025 (no additional eligible studies were identified).

### Evidence acquisition

***Search strategy*** - A comprehensive literature search was conducted collaboratively by an experienced librarian and two of the authors. No publication year limit was set. Diagnostic validation studies, whether prospective or retrospective, published in English, were eligible for inclusion. The focus was on investigations reporting on the performance of clinical examination, mammography, AUS, or MRI in detecting axillary lymph node metastases in patients with early-stage breast cancer, defined as cancer found only in the breast or nearby lymph nodes that has not spread to other parts of the body. Emphasis was placed on studies involving women with early-stage breast cancer who were overweight or obese women or were stratified by BMI. No minimum number of overweight or obese patients was established. The pathology result was taken as the gold standard for the assessment of lymph nodes obtained through SLND or ALND. Studies that evaluated accuracy in the general breast cancer population were included only if they also reported on accuracy in overweight or obese women or classified performance findings according to BMI.

***Database search*** - We used controlled vocabulary (Medical Subject Headings, Excerpta Medica Tree descriptors, and Health Sciences Descriptors of the Brazilian Virtual Library of Health) and free-text terms covering four concept blocks: breast cancer; axillary lymph nodes; index tests (clinical examination, ultrasonography, mammography, and MRI); and obesity/body mass index. During the peer-review process, one potentially relevant study-Macaione et al.^**(^25^)**^-was suggested by a reviewer. After assessing its eligibility according to our predefined inclusion criteria, this study was incorporated into the final analysis, bringing the total number of included studies to nine.

**Figure 2:**
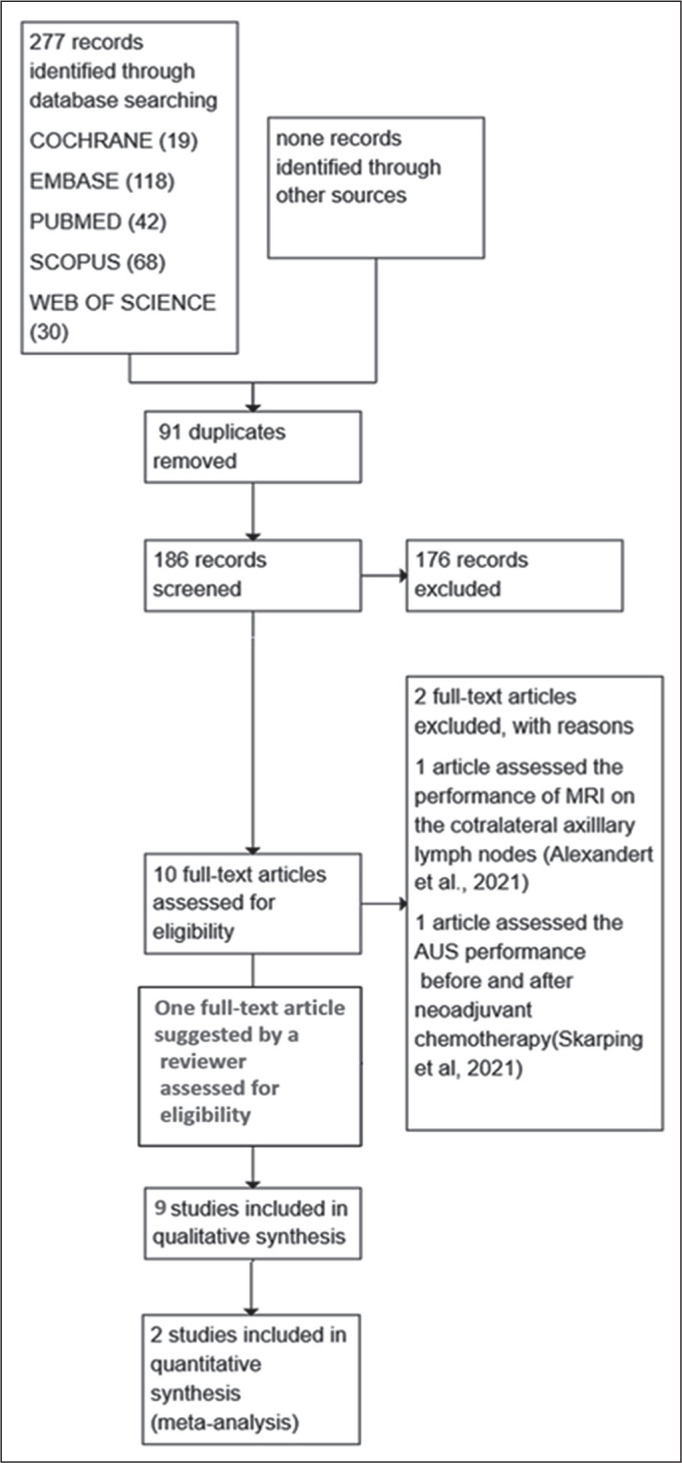
Flow chart of the evidence acquisition and synthesis process for the systematic review and meta-analysis. A total of 277 records were identified through searches of the Cochrane, Embase, PubMed/Medline, Scopus, and Web of Science databases. After removal of 91 duplicates, 186 records were screened, of which 176 were excluded. Ten full-text articles were assessed for eligibility, and two were excluded with reasons. One additional full-text article suggested by a reviewer-Macaione et al.^(^25^)^-was assessed for eligibility and included. Therefore, nine studies were included in the qualitative synthesis and two were included in the quantitative synthesis (meta-analysis).

***Study management*** - Retrieved records were imported into Rayyan software (Qatar Computing Research Institute, Doha, Qatar) for efficient management^**(^26^)**^. Figure 2 illustrates the article selection process. After eliminating duplicates, two independent reviewers screened the remaining studies by reviewing titles and abstracts. Initially included studies underwent further scrutiny with full-text reviews to confirm eligibility. Case reports were excluded, as were animal studies and conference abstracts, as well as studies involving patients with locally advanced breast cancer, with or without neoadjuvant chemotherapy, those in which CT, PET/CT, or lymphoscintigraphy was the only imaging modality, those in which the sample was not restricted to breast cancer patients, and those involving male breast cancer patients. Disagreements were resolved through consensus.

***Data extraction*** - The same two researchers systematically extracted data from the included studies. Utilizing Review Manager Web (RevMan Web) version 6.6.0 (The Cochrane Collaboration, 16 Nov 2023), we ordered the articles from study one to study nine, reflecting their order of inclusion in RevMan Web^**(^6^,^16^,^19^,^25^,^27^-^31^)**^. Extracted data included first author, year, journal, data source, journal article reference, patient sampling, patient characteristics, setting, index test, target condition, reference standard, flow and timing, covariates, and outcome measures (true positives, false positives, false negatives, and true negatives). Although most of the studies lacked raw data on outcome measures, they were included for qualitative assessment.

***Quality assessment*** - The quality of the studies underwent evaluation using the Quality Assessment of Studies of Diagnostic Accuracy 2 (QUADAS-2) tool included in a systematic review^**(^24^)**^. This tool comprises four domains: patient selection; index test; reference standard; and patient flow and timing of index and reference tests. All of these domains were assessed in terms of the risk of bias, and the first three domains were also evaluated in terms of applicability concerns.

In the risk of bias assessment sessions, signaling questions were employed to evaluate the risk of bias in each study. When all the answers for a specific domain were “Yes”, the risk of bias was classified as low. However, if at least one of the questions was answered with a “No”, the study was considered to have potential for bias, being classified as high risk. When the available data were insufficient to allow a conclusive judgment, the level of risk was classified as “unclear”.

**Figure 3:**
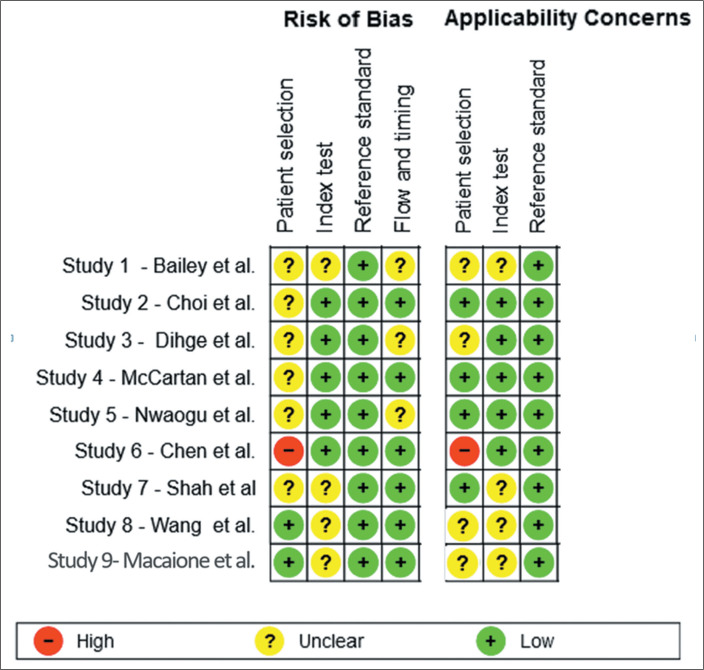
Summary of risk of bias and applicability for each study, showing the authors’ judgments across each domain, with a description of each article listed by the lead author’s name. Traffic-light plot summarizing the risk of bias and applicability concerns for each included study according to the QUADAS-2 tool result. The domains assessed were patient selection, index test, reference standard, and flow and timing.

**Figure 4:**
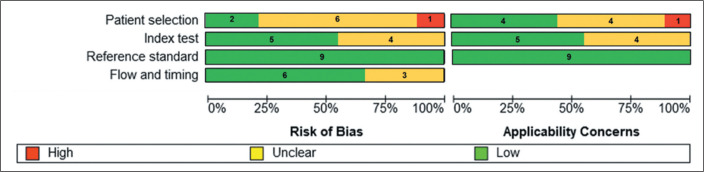
Summary of the quantitative analysis of the risk of bias and applicability assessments of the studies across the evaluated domains. Summary bar plot of QUADAS-2 assessments across all nine included studies, showing the proportions of low risk, unclear risk, and high risk judgments for each domain: patient selection, index test, reference standard, and flow and timing.

The applicability sessions followed a structure similar to that of the risk of bias sections but did not include the signaling questions. In these sessions, we recorded the information that supported the applicability judgment and subsequently classified the level of concern regarding whether the study matched the central question of the review. These findings are presented in Figures 3 and 4, the first providing a description of each article by the name of the lead author and the second showing the number of studies in each score category.

### Evidence synthesis

A total of nine studies met the eligibility criteria and were included in the analysis. These studies primarily investigated the diagnostic performance of different methods for preoperative axillary staging in early-stage breast cancer, with a focus on overweight or obese women. Six of the nine studies evaluated AUS, two of these also included the combination of AUS with fine-needle aspiration (AUS + FNA or core needle biopsy). One study focused exclusively on clinical examination, another focused on MRI, and another assessed mammography performance. These findings are summarized in Table 1.

**Table 1:** Variables of interest in the selected studies.

Study	First author	Year	Country	Journal of publication	Index test(s)	Design	Main features of the evaluated population	Body weight groups [BMI in kg/m^2^] (N or n)	Statistical analysis
1	Bailey^(^16^)^	2015	USA	The American Surgeon	AUS	Diagnostic performance retrieved from a institutional cancer registry	Patients with clinically node-negative axilla	All weights (249)	BMI as a continuous variable: binary logistic regression model
2	Choi^(^19^)^	2012	Korea	Ultrasound in Medicine & Biology	AUS	Diagnostic performance retrieved from a database	Patients with negative results on AUS	Overweight (121) Obese(17)	FN results compared between BMI < 25 and BMI > 25: chi-square test
3	Dihge^(^27^)^	2016	Sweden	Acta Oncologica	AUS AUS + FNA	Diagnostic performance retrieved from a database Diagnostic performance retrieved from a database	Patients who underwent AUS Patients with suspicious AUS who underwent AUS-guided FNA biopsy	All weights (473) (< 30:378)/(> 30: 95) All weights (45) (< 30: 30)/(> 30:12)	Sensitivity, specificity, PPV, and NPV
4	McCartan^(^28^)^	2016	USA	Surgical Oncology	Clinical examination	Diagnostic performance retrieved from a database	Patients with clinically node-negative axilla	All weights (5,262) (< 25.0: 2,165)/ (25.0-29.9:1,606)/ (> 30.0:1,491)	BMI as a continuous variable: Wilcoxon rank-sum test BMI as categorical variable: TN and FN
5	Nwaogu^(^29^)^	2015	USA	Journal of Surgical Research	AUS	Diagnostic performance retrieved from a database	Patients with normal AUS	All weights (118)	TN vs. FN, with BMI as a continuous variable: two-sample t-test or Mann- Whitney U test
6	Chen^(^30^)^	2022	Taiwan	World Journal of Surgical Oncology	MRI	Diagnostic performance retrieved from a database	Patients who underwent preoperative MRI	Underweight: < 18.5 (71)/ Normal weight: 18.5-24.0 (1,045)/ Overweight: 24.1-26.9 + obese: > 27.0 (968)	Association between BMI and the evaluation of ALNM on MRI: chi-square test
7	Shah^(^31^)^	2014	USA	Surgical Oncology	AUS	Diagnostic performance retrieved from a database	Patients who underwent AUS	Normal: < 25 (456) / Overweight: 25.0-29.9 (427) / Obese: > 30 (492)	Likelihood ratio for categorical data: chi-square test
8	Wang^(^6^)^	2023	China	Clinical Breast Cancer	MG	Prospective	Patients with early-stage breast cancer	Underweight/lean: < 25 (51/20) Overweight and obese: > 25 (24)	Intergroup differences: Student’s t-test Diagnostic accuracy: SROC curves and areas under the curve
9	Macaione^(^25^)^	2020	Italy	Anticancer Research	AUS	Retrospective cohort	Patients with clinically node-negative axilla who underwent SLNB	Normal/underweight: < 25 (62) Overweight: 25-30 (46) Obese: > 30 (36)	FN rates and NPVs between BMI groups: chi-square test Correlation between BMI and SLNB positivity: Spearman's test

PPV, positive predictive value; NPV, negative predictive value; FN, false negative; TN, true negative; ALNM, axillary lymph node metastasis; MG, mammography; SLNB, sentinel lymph node biopsy.

Seven of the nine studies (studies 1-7) were based on data retrieved from large institutional or national databases. However, all of them presented an unclear risk of bias in patient selection, often due to retrospective designs, the exclusion of incomplete records, or the retrospective reclassification of axillary status from imaging archives. In a study evaluating clinical examination, for example, 25% of the participants had already undergone AUS prior to physical examination, potentially influencing the clinical assessment (Figures 3 and 4). Study 9 was a retrospective single-center cohort study conducted by Macaione et al.^**(^25^)**^. Only one study (study 8) enrolled patients prospectively^**(^6^)**^.

Among the studies evaluating AUS was study 1, conducted by Bailey et al.^**(^16^)**^, for which the risk of bias and degree of applicability were determined to be unclear because of the retrospective assessment of ultrasound results and the absence of clearly defined criteria for identifying abnormal lymph nodes. Another major limitation of that study was the prolonged interval between AUS and definitive axillary staging, often exceeding 30 days, which was judged to be a relevant concern in the flow and timing domain of the QUADAS-2 assessment (Figure 3). That delay could have allowed disease progression and consequently affected diagnostic accuracy.

In study 3, conducted by Dihge et al.^**(^27^)**^, the main limitations were related to the high proportion of patients with micrometastatic disease, which may not be detectable by AUS or AUS + FNA. In addition, 18% of the cases with suspicious AUS findings were not evaluated with FNA, without adequate explanation, raising the possibility of an index test bias.

In study 5, conducted by Nwaogu et al.^**(^29^)**^, an unclear risk of bias in flow and timing was attributed to a significant time gap between imaging and surgery-averaging 67 days for non-obese patients and 26 days for obese patients-potentially affecting diagnostic accuracy.

Study 6, conducted by Chen et al.^**(^30^)**^, which evaluated MRI, was the only one to receive a high risk of bias and high level of concern regarding applicability. This was due to the use of Taiwanese BMI thresholds for classifying patients as overweight and obese, as well as the presence of significant differences in age and tumor size across BMI groups, which could have affected MRI performance.

Study 7, conducted by Shah et al.^**(^31^)**^ was also assigned an unclear level of concern regarding the index test. In that study, ultrasound-guided core needle biopsy (with a 14- or 18-gauge needle) was performed at the discretion of the radiologist and was not standardized across patients, limiting the reliability of results.

Among all of the studies included, only study 8, conducted by Wang et al.^**(^6^)**^, had a prospective design. It was a pilot study evaluating a novel mammographic technique, known as the two-dimensional (2D)-axilla view, in a cohort of 75 patients with early-stage breast cancer. It also assessed the conventional mediolateral oblique and tomosynthesis-three-dimensional (3D)-axilla-views. Despite that innovative approach, the limited sample size raised concerns about generalizability and applicability of findings.

In study 9, conducted by Macaione et al.^**(^25^)**^, the risk of bias and level of concern regarding applicability were both judged to be unclear for the index test. Although AUS was combined with tissue sampling, it was not specified under which circumstances FNA or core needle biopsy was performed, which limits reproducibility. In addition, the criterion of loss of ovality as predictive of nodal involvement was adopted without a supporting reference and in the absence of clearly defined criteria for abnormal lymph nodes, further increasing the level of uncertainty.

**Figure 5:**
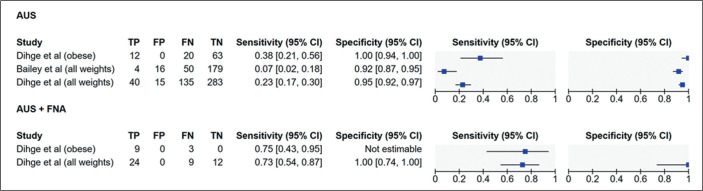
Forest plot of sensitivity and specificity of AUS and AUS + FNA for the detection of axillary metastasis in all-weight or obese patients with early-stage breast cancer. The plots display individual study estimates with 95% confidence intervals for sensitivity (left) and specificity (right). Results are shown separately for AUS alone and AUS + FNA. (TP, true positive; FP, false positive; FN, false negative; TN, true negative).

**Figure 6:**
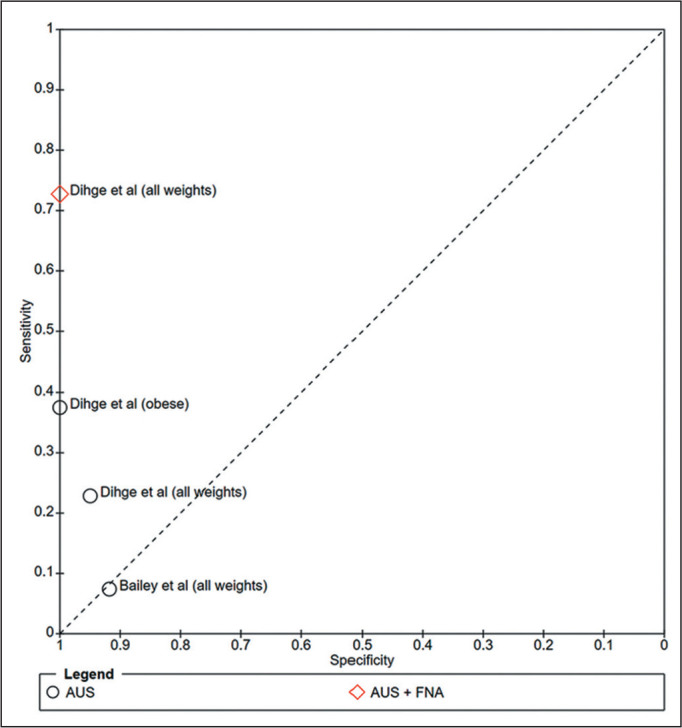
SROC curves of AUS and AUS + FNA for the detection of axillary lymph node metastasis in overweight or obese patients with early-stage breast cancer. Each point represents the diagnostic performance, in terms of sensitivity and specificity, in each study. The SROC curves illustrate the trade-off between sensitivity and specificity across studies. The AUS and AUS + FNA results are plotted separately for obese and general populations when available. (Dihge et al.^(^27^)^ and Bailey et al.^(^16^)^).

Regarding the availability of quantitative diagnostic data, only Dihge et al.^**(^27^)**^ reported complete 2×2 contingency table data (true positives, false positives, false negatives, and true negatives) stratified by weight group (“all weights” and “obese”). Bailey et al.^**(^16^)**^ also provided raw diagnostic data, although only for the general population (“all weights”). These studies enabled the construction of forest plots illustrating sensitivity and specificity for AUS and AUS + FNA (Figure 5). Summary receiver operating characteristic (SROC) curves were generated to visually assess the heterogeneity of sensitivity and specificity across studies (Figure 6). The degree of statistical heterogeneity was moderate to substantial, with an *I^^2^^* value of 73% and a between-study variance (*T^^2^^*) of 2.51. This level of inconsistency precluded the combination of data into a single pooled estimate or quantitative meta-analysis. Table 2 summarizes the conclusions drawn from each study and highlights the consistency of results.

**Table 2:** Results regarding the performance of methods for the preoperative detection of axillary lymph node metastasis in relation to the baseline weight or BMI of the patients.

Study	Result
1 - Bailey etal.^(^16^)^	Obesity (BMI > 30 kg/m^2^) was not significantly associated with overall accuracy/concordance of the AUS examination.
2 - Choi et al.^(^19^)^	There was no significant difference between the normal weight (BMI < 25 kg/m^2^) and overweight (BMI > 25 kg/m^2^) groups in terms of the number of false-negative AUS results.
3 - Dihge et al.^(^27^)^	There were no BMI-related differences in the per-formance of AUS + FNA on preoperative axillary nodal staging.
4 - McCartan et al.^(^28^)^	Elevated BMI was not associated with a higher likelihood of sentinel lymph node positivity or heavy nodal disease burden among women staged as negative on physical examination.
5 - Nwaogu et al.^(^29^)^	An association between higher BMI and false-negative AUS results was not supported by the analyses.
6 - Chen et al.^(^30^)^	The accuracy of MRI in predicting axillary lymph node metastasis was lower in overweight women than in normal and underweight women.
7 - Shah et al.^(^31^)^	The sensitivity of preoperative AUS for detect-ing nodal metastasis was comparable between obese and nonobese patients, whereas its speci-ficity was better in obese patients.
8 - Wang et al.^(^6^)^	For 2D-axilla, 3D-axilla, and 2D mediolateral oblique mammography, the areas under the curve were higher in the overweight/obese group than in the underweight/lean group, although the differences were not significant.
9 - Macaione et al.^(^25^)^	The NPV of preoperative AUS was found to be sig-nificantly influenced by the quantity of adipose tissue.

## DISCUSSION

### Principal findings

This systematic review identified a consistent trend across the included studies suggesting that a high BMI does not impair the diagnostic performance of methods for preoperative detection of axillary lymph node metastases in patients with early-stage breast cancer. Although the number of available studies was limited and most were marked by some degree of methodological concern, the convergence of findings strengthens the overall evidence base. In particular, AUS, with or without FNA, demonstrated stable diagnostic performance across different BMI categories, indicating that there is no need for adjustments in technique or interpretive criteria for overweight or obese patients. One exception is the retrospective cohort study conducted by Macaione et al.^**(^25^)**^, who reported that the negative predictive value (NPV) of AUS decreased in parallel with an increase in BMI; that finding is addressed below.

**Figure 7:**
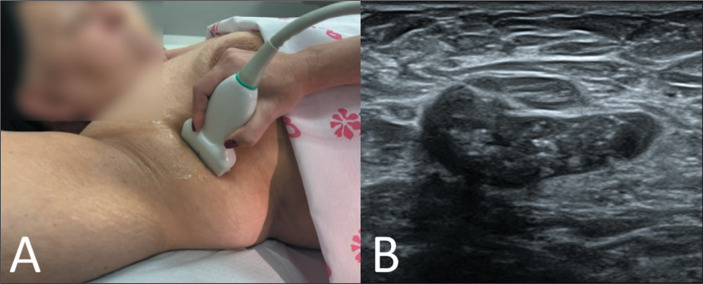
AUS technique and corresponding imaging findings in an obese patient. A: Proper transducer-skin contact, facilitated by axillary cavity filling with subcutaneous fat, potentially improving image quality. B: Ultrasound image showing cortical thickening, effacement of the fatty hilum, and calcifications-findings suggestive of metastatic lymph node involvement. Despite the thickness of the subcutaneous fat layer, image acquisition and interpretation were not impaired.

Two hypotheses may explain why AUS performance remains robust in overweight and obese patients. First, the increased thickness of the subcutaneous fat layer may not cause sufficient attenuation of the ultrasound signal to significantly degrade image resolution. In addition, the presence of fat in the axillary cavity may actually enhance acoustic coupling between the transducer and the skin surface, improving image acquisition. This improved transducer adherence might account for the increased specificity of AUS in obese patients, as reported by Shah et al.^**(^31^)**^. Figure 7 illustrates this enhanced contact and the preservation of sonographic detail, even in the presence of fatty tissue. That notwithstanding, Macaione et al.^**(^25^)**^ observed that the NPV of AUS was lower in the patients with higher BMI. Those authors used nonstandardized AUS criteria and did not prespecify when tissue sampling (FNA/core needle) should be performed, features that may introduce spectrum and verification bias, thus limiting reproducibility. Differences in case mix and retrospective design likely contributed to the discrepancy with the remaining literature. Taken together, these data support cautious interpretation of a negative AUS in obese patients and underscore the need for standardized AUS criteria and prospective, BMI-stratified studies.

Although only one study specifically evaluated clinical examination^**(^28^)**^, its large sample size-encompassing 5,262 patients, including 1,491 obese women-exceeded the total combined sample size of all of the other studies included in this review. It is notable that the authors of that study reported no significant association between BMI and the accuracy of the physical examination in identifying axillary lymph node metastases. One plausible explanation is that, despite excess adipose tissue, the soft consistency of fat may not preclude palpation of the underlying nodal structures, maintaining the effectiveness of the clinical examination. In contrast, mammography has traditionally been considered suboptimal for axillary assessment due to its limited field of view and low spatial resolution for structures beyond the breast parenchyma. However, Wang et al.^**(^6^)**^ evaluated a novel mammographic view-the 2D-axilla view-designed specifically to improve visualization of the axilla. In this pilot study, the axilla view provided broader coverage than the conventional mediolateral oblique view and was assessed alongside digital (3D) tomosynthesis. Although not statistically significant, the area under the SROC curve was consistently higher in the overweight and obese subgroup across all mammographic views, suggesting a potential positional advantage in this population. Nevertheless, the small sample size and exploratory nature of the study call for further investigation to validate these findings.

Among the studies that evaluated MRI, only one^**(^30^)**^ reported lower diagnostic performance in overweight women. However, that study was rated as having a high risk of bias and a high level of concern regarding applicability, mainly due to the use of regional BMI cutoffs and an imbalance between groups in terms of patient characteristics. As such, this isolated finding should be interpreted with caution. Additional prospective studies with samples that are larger and more representative are needed in order to confirm or refute the impact of obesity on MRI performance in this context.

### Strengths

To our knowledge, this was the first systematic review and meta-analysis to evaluate the diagnostic performance of clinical examination and the primary imaging modalities-including AUS, mammography, and MRI-in the preoperative detection of axillary lymph node metastasis specifically in overweight and obese women with early-stage breast cancer. By focusing on this underrepresented subgroup, our study addresses an important gap in the literature and provides clinically relevant insights for improving axillary staging without the need for invasive procedures. In addition, the use of a comprehensive, structured approach to article selection, quality appraisal with the QUADAS-2 tool, and adherence to the PRISMA-DTA guidelines ensured methodological transparency and reproducibility.

### Limitations

Despite the methodological rigor adopted in this review, several limitations must be acknowledged. First, the number of eligible studies was small (n = 9), and none were designed specifically to evaluate diagnostic performance by BMI category. Instead, subgroup analyses or stratifications were performed post hoc or reported incidentally, often without adjustment for confounders such as age, tumor size, or histologic subtype. In addition, all included studies presented some risk of bias or applicability concerns, especially in the domains of patient selection and index test interpretation. Only one study provided complete 2×2 contingency table data stratified by BMI/weight group, whereas another reported raw diagnostic data only for the overall cohort, limiting our ability to perform a robust quantitative meta-analysis. Although forest plots and SROC curves were generated for AUS and AUS + FNA, the observed statistical heterogeneity (*I^^2^^* = 73%) further discouraged data pooling. Furthermore, the lack of standardization across studies in terms of imaging protocols, operator expertise, and definitions of abnormal lymph nodes may have introduced further variability, including nonstandard AUS criteria in some cohorts, such as that evaluated in the Macaione et al.^**(^25^)**^ study. Another important limitation is the potential selection bias in retrospective studies relying on institutional databases, in which evaluators interpreting the images may not have been blinded to clinical characteristics or surgical outcomes. Finally, differences in BMI classification thresholds across countries, as evidenced by the MRI study conducted in Taiwan^**(^30^)**^, limit the generalizability of some findings and highlight the need for harmonized criteria when comparing international data.

### Implications and future directions

Taken together, the findings of this review suggest that standard clinical and imaging tools-particularly AUS and physical examination-retain their diagnostic value in overweight and obese patients without a need for technical adjustments. One exception is the retrospective cohort study conducted by Macaione et al.^**(^25^)**^, who reported an inverse correlation between the NPV of AUS and patient BMI. That indicates a need for caution when interpreting a negative AUS result in obese patients and for the use of standardized AUS criteria in future studies. However, the limited number and quality of studies underscore the need for further prospective, BMI-stratified diagnostic studies using standardized imaging criteria and clearly defined outcome measures. Future research should also explore whether emerging techniques, such as contrast-enhanced ultrasound or radiomics-based MRI interpretation, offer additional advantages in this population.

## CONCLUSION

This systematic review suggests that fundamental components of axillary staging in early-stage breast cancer-namely, physical examination and AUS-maintain satisfactory diagnostic performance in overweight and obese women. Notably, one study reported that the NPV of AUS was lower when BMI was higher^**(^25^)**^, which warrants cautious interpretation of negative AUS findings in this subgroup. Despite longstanding concerns about the potential negative impact of obesity on clinical and imaging assessments, current evidence does not support the need for routine technical modifications or adjusted interpretive criteria. Nevertheless, these conclusions should be interpreted cautiously given the limited number of studies, heterogeneity of methods, and common methodological limitations. As such, our findings underscore the need for further, high-quality, prospective research specifically designed to evaluate the diagnostic performance of axillary staging tools across different BMI categories, with standardized definitions and protocols.

## Data Availability

Not applicable.
